# Social inequalities and health inequity in Morocco

**DOI:** 10.1186/1475-9276-5-1

**Published:** 2006-03-07

**Authors:** Abdesslam Boutayeb

**Affiliations:** 1 Département de Mathématiques et Informatique Faculté des Sciences – Université Mohamed I^er ^Oujda, Morocco

## Abstract

**Background:**

According to the last census, Morocco has a population approaching 30 million people. The country has made good progress in the control of preventable childhood diseases but social inequalities and health inequities remain major problems for the third millennium. Despite the progress achieved during the last decade, the country still ranks at the 125^th ^place according to the Human Development Index. This unpleasant position is mainly explained by illiteracy, education and health indicators.

**Method:**

Our study was based mainly on annual reports and regular publications released by the United Nations (UN), United Nations Development Programme (UNDP), World Health Organisation (WHO), The Moroccan Health Ministry and related papers published in international journals.

**Results and discussion:**

As indicated by the last Arab Human Development Reports (AHDR 2002, AHDR 2003, AHDR 2004) and implicitly confirmed by the "National Initiative for Human Development" (NIHD) launched in May 2005 by the King of Morocco, many districts and shanty towns, urban or peri-urban, and a multitude of rural communes live in situations characterized by difficult access to basic social services of which education and health are examples.

**Conclusion:**

Recent evidence showed that improved health is more than a consequence of development. It is a central input into economic and social development and poverty reduction. Serious initiatives for human development should consider the reduction of social inequalities and health inequities as a first priority. Otherwise, the eventual development achieved cannot be sustained.

## Background

According to the last census, Morocco has a population approaching 30 million people, experiencing a transition on different levels. In 2005, 55% of the population is living in urban areas, compared to 43% in 1982 and 29% in 1960. The Moroccan population is young, with 38% under the age of 14 years, and life expectancy at birth has increased from 65 in 1980 to 68.5 in 2004. The country has made good progress in the control of preventable childhood diseases but social inequalities and health inequities remain the major problems for the third millennium.

Despite the diverse resources (agriculture, phosphates, fishing, potentialities for tourism, etc...) and the progress achieved during the last decade, the country still ranks 125^th ^according to the Human Development Index (HDI) (UNDP, 2004)[1]. This unpleasant position is mainly explained by low income, high adult illiteracy, lack of generalized education, and health indicators. The reports released by the UNDP and essentially the last three Arab Human Development Reports (AHDR2002-2004) [2-4] indicate that little improvement has been achieved during the last decade. The Moroccan authorities have been trying to find excuses rather than dealing with the real causes of such insufficiencies in human development. The "National Initiative for Human Development" (NIHD), launched in May 2005 by the King of Morocco, has at last, admitted that many districts and shanty towns, urban or peri-urban, and a multitude of rural communes live in uncomfortable situations characterized by difficult access to basic social services such as education and health.

The Moroccan system of health care production is organized into three sectors:

• The public health care sector: This is the largest sector present throughout the country, providing 85% of the country's hospital beds and representing the main employer of health professionals in Morocco. This sector is supposed to deal particularly with the needs of poor and rural populations who are unable to afford the service offered by the private sector.

• The private health care sector: This is a profit-making sector which is mainly present in cities. It is principally attended by people with sufficient income or those who have health insurance.

• The non-profit health sector: This sector is present exclusively in big cities and run mainly by the National Security Fund (CNSS). Its care is devoted to the 16% of the population covered by health insurance.

During the last decades, an important literature was devoted the differences and connections existing between social justice and health equity [5-8]. More and more publications are dealing with health of the poor [9], health equity and health as a cornerstone of sustainable development [10]. According to the authors of Dying for Growth [11], economic growth, far from being a panacea, often accelerates the suffering of poor and marginalized people. Building on this, the present paper deals with social inequalities and health inequity in the special case of Morocco.

## Method

Data from different sources were used in order to study the inequalities in terms of income, consumption, education and health opportunities. In our search, we used MEDLINE data base (via the Pub Med web site) for related publications, the reports released by The World Health Organisation (WHO) the United Nations Development Programme (UNDP) and regular publication of the Moroccan health authorities.

## Results and discussion

Morocco is characterized by contrasting phenomena and huge inequalities at different levels. For ease of clarity, we will concentrate on disparities (milieu, sex, region) according to income, education and health.

### Stagnant growth and income inequality

As indicated by the 2002 Arab Human Development Report [2], the per capita income in Morocco is growing very slowly indicating a quasi-stagnation of economic growth and a relative deterioration in the average standard of living for the whole population. This global problem of low income is exacerbated by bad governance and huge inequalities. Indeed, the ratio (11.7) of the richest 10% to the poorest 10% is excessively high compared with developed countries and even with developing countries (Table 1). As a corollary, 19% of the Moroccan population live under the national poverty line, of which 70% are living in rural area, yielding a second discrimination at a regional level. The estimated earned income of women is less than 40% of that of men and the share of non-agricultural wage employment is 73% for men and 27% for women. Consequently, the discrimination encountered by a poor rural woman is threefold. The inequality is found even in the same administration sector. For instance, the salary of a primary teacher represents just 4% of the salary of a president of a university.

**Table 1:** Share of income & consumption: Ratio of the richest 10% to the poorest 10% [4, 19]

Country	Ratio	Country	Ratio
USA(2000)	5.43	Algeria(2002)	9.6
UK(1999)	4.54	Egypt(2002)	8.0
Holland (1999)	3.27	Jordan(2002)	9.1
Germany (2000)	3.17	Morocco(2002)	11.7
Sweden (2000)	2.95	Tunisia(2002)	13.4
Finland (2000)	2.90	Yemen(2002)	8.6

After 50 years of independence, 50% of the Moroccan population aged 15 and above are illiterate with women representing nearly 2/3. As showed in Table 2, very few Arab countries do worse than Morocco. Knowing that illiteracy rates in the Arab world remain higher than the international average and are even higher than the average in developing countries, it is clear that the burden of illiteracy will continue to affect the human development index of Morocco for a while.

**Table 2:** Adult literacy rate (% ages ≥ 15) and combined gross enrolment ratio [4]

Country	HDI	Literacy (%)	Literacy (%)	Enrolment (%)	Enrolment (%)
	2002 Rank	Male	Female	Male	Female
Algeria	108	78.0	59.6	72.0	69.0
Bahrain	40	91.5	84.2	77.0	82.0
Comoros	136	63.5	49.1	50.0	41.0
Djibouti	154	76.1	55.5	28.0	20.0
Egypt	120	67.2	43.6	80.0	72.0
Jordan	90	95.5	85.9	76.0	77.0
Kuwait	44	84.7	81	71.0	81.0
Lebanon	80	92.4	81	77.0	79.0
Lybia	58	91.8	70.7	93.0	100.0
Mauritania	152	51.5	31.3	46.0	42.0
**Morocco**	**125**	**63.3**	**38.3**	**61.0**	**52.0**
Oman	74	82.0	65.4	62.0	63.0
Qatar	47	84.9	82.3	79.0	84.0
Saudi Arabia	77	84.1	69.5	58.0	57.0
Sudan	139	70.8	49.1	39.0	34.0
Syria	106	91.0	74.2	62.0	57.0
Tunisia	92	83.1	63.1	74.0	75.0
UAE	49	80.7	75.6	65.0	72.0
Yemen	149	69.5	28.5	66.0	37.0

A similar statement can be made for the combined gross enrolment rate in primary, secondary and tertiary schools (Table 2 columns 3&4). Once more, Morocco is seven points below the average of the Arab countries (57 vs. 64). As stressed by the 2002 Arab Human Development Report [2], Morocco and the Arab countries are not expected to catch up with the industrialized countries' mid-1990s enrolment levels for all three levels of education before 2030!

Moreover, for literacy and enrolment, the gap between Moroccan rates and the Arab world average is much accentuated for women (24.5 and 10.8 respectively) than for men (15.7 and 4 respectively).

In general, as indicated in Table 3 for literacy, Moroccan inequality is at least a three-dimensional space, spanned by Milieu, Sex and Income class.

**Table 3:** Literacy rate 1998–1999: Milieu, sex and class of income [20]

Category	Male Urban	Male Rural	Female Urban	Female Rural
Richest 20%	87.6	62.6	63.6	21.8
Poorest 20%	64.4	43.9	38.9	11.9

### Health inequity

In Morocco, until recently, health was considered as a non-productive sector. Indeed, during the last four decades, the budget affected to health represented, on average, 1% of GDP each year and bad governance conducted to an ill-health system. Consequently, by the dawn of the third millennium, only 40% of births are attended by skilled health personnel, many women continue to die during childbirth and infant mortality rate remains relatively high Table 4). With an average of one doctor for 2100 inhabitants, Morocco compares badly with countries of equivalent level of development. Moreover, the partition is unfair since the numbers vary from one doctor for 840 inhabitants in one region to one doctor for 4600 in another region (Table 5). Inequities are also found between cities and rural areas, with nearly 30% of the rural population living at a distance of 10 km from any health facility. More generally, the global health expenditure is very low and mainly supported by households (60%), whereas 40% of all expenditure engaged by the health ministry goes to the richest 20% of the population [12]. Health insurance covers only 16% of the population and nearly exclusively in cities. This percentage is expected to increase to 32% of the population under the new law on Compulsory Health Insurance supposed to be applicable by 2006. According to the World Health Organisation and using the Health Adjusted Life Expectancy at birth (HALE) which excludes years of illness, the expectation of lost healthy years at birth is 11.9 years for women vs. 9.4 for men, which shadows the relative high rates of life expectancy [2] (Table 6). In 1999, the percentage of the population with sustainable access to affordable essential drugs was estimated to be between 50 and 79%. A more precise estimate was given by the national survey on living standards conducted by the statistical department of the planning ministry in 1998–99 concluding that more than 33% of the population is unable to afford necessary medical care. The inequity is more exacerbated in rural areas where this percentage reaches 44 % (Table 7). Inequities also exist for the free access to public structures since the richest are more likely to get free care than the poorest [11]. Households devote on average 6.5% of their budget to health care. This percentage becomes 9% for the poorest 20% whereas it represents 3.9% for the richest 20%. With 19% of the population living under the threshold of poverty and the consequences of a multi-dimensional transition, health care is becoming less and less affordable. For instance, the cost of care and treatment for an insulino-dependent diabetic person is estimated at USD120 per month [13]. This is nearly the monthly wages of a non-qualified manual worker, and this is not to mention the cost of treatment for other conditions like heart disease, kidney failure and cancer.

**Table 4:** Health indicators: Morocco compared to Arab countries [4]

Country	Life Expectancy at birth (years)		Expectation of Lost healthy years		Maternal Mortality Ratio per 100 000	Infant Mortality Rates per 1000 live births	Physicians Per 100 000 people
	Female	Male	Female	Male			
	2002		2002		2000	2002	1990–2003
Algeria	71.1	68.0	09.6	07.9	140	39	85
Bahrain	75.8	72.4	10.1	07.9	28	13	169
Comoros	62.0	59.2	09.6	07.8	480	59	7
Djibouti	47.0	44.8	07.4	06.1	730	100	13
Egypt	70.8	66.6	08.8	07.4	84	35	218
Jordan	72.4	69.6	10.9	09.0	41	27	205
Kuwait	78.9	74.8	10.6	08.2	5	9	160
Lebanon	75.0	71.8	10.4	08.4	150	28	274
Libya	75.3	70.7	10.5	08.1	97	16	120
Mauritania	53.9	50.7	08.2	06.9	1000	120	14
**Morocco**	**70.3**	**66.6**	**11.9**	**09.4**	**220**	**39**	**49**
Oman	74.3	70.9	11.1	08.3	87	11	137
Qatar	75.3	70.4	10.0	08.2	7	11	220
Saudi Arabia	73.6	71.0	11.0	08.6	23	23	153
Sudan	57.0	54.1	09.4	07.8	590	64	16
Syria	73.0	70.5	10.5	08.5	160	23	142
Tunisia	74.8	70.7	10.3	08.2	120	21	70
UAE	77.3	73.2	10.9	07.8	54	8	177
Yemen	60.9	58.7	11.5	10.8	570	79	22

**Table 5:** Geographical disparities [12]

Region	HDI Education	Inhabitants per physician	Inhabitants per dentist	HDI Global
Taza-AlHoceima-Taounate	0.337	4587	62034	0.541
Marrakech-Tensift-Al Haouz	0.394	3329	29404	0.565
Doukkala-Abda	0.405	3738	28909	0.566
Gharb-Chrarda-BeniHssen	0.426	2822	20871	0.574
Tadla-Azilal	0.427	4084	47200	0.577
Tanger-Tétouan	0.455	2611	21443	0.588
Souss-Massa-Draa	0.462	3539	32256	0.594
Oriental	0.480	2459	14646	0.598
Fès-Boulemane	0.486	2303	11321	0.598
Chaouia-Ouardigha	0.493	2750	27033	0.604
Meknès-Tafilalet	0.572	2729	23425	0.623
Guelmim-EsSemara	0.572	1918	104250	0.649
Rabat-Salé-Zemmour-Zaer	0.614	836	4769	0.660
Grand Casablanca	0.697	999	3870	0.697
Oued Ed-Dahab-Lagouira	0.739	1918	23000	0.740
Laâyoune-Boujdour-Sakia-El Hamra	0.768	1918	22444	0.742
Total National	0.495	2084	12495	0.607

**Table 6:** Gender inequalities in health, income and education [4]

	Male	Female
Life expectancy at birth (2002) in years	66.6	70.3
Expectation of lost healthy years at birth (2002) in years	9.4	11.9
Estimated earned income (2002) (PPP US$)	5,354	2,153
Share of non-agricultural wage employment 2001 (%)	73	27
Combined gross enrolment ratio for primary, secondary and tertiary level schools 2001/02 (%)	61	52
Adult literacy rate (2002) ages 15 and above (%)	63.3	38.3

**Table 7:** Regional inequalities [4, 12]

	Urban	Rural
% of population below national poverty line 1992–2000	12.0	27.2
% of population using improved drinking water source 2000	98	56
% of population using adequate sanitation facilities 2000	86	44
% of population unable to afford essential medical care in 1998–99	28.6	44

With these inequities and such an ill-health system, Morocco is facing the double burden of communicable and non-communicable diseases [14-16].

Non-communicable diseases (NCDs) are responsible for 55.8% of the total Disability Adjusted Life Years (DALYs). Cardio-Vascular Diseases, cancer, respiratory diseases and diabetes are the main contributors. The prevalence of the main cardiovascular risk factors was considered in a study conducted in 2000 on a representative sample aged 20 years and over [17]. According to this study, the prevalence of hypertension, hyper-cholesterolaemia and diabetes were respectively 33.6%, 29% and 6.6%. The prevalence of obesity was markedly higher in females in urban areas with 40% of women overweight and an average body mass index (BMI) of 25.6 kg/m^2 ^versus 23.8 kg/m^2 ^for males.

The incidence and mortality of cancer are increasing, especially for breast, lung, cervical, colorectal and stomach cancer which represent altogether 42% of all new cases and 44% of all deaths caused by cancer.

The morbidity caused by Communicable Diseases (CDs), perinatal and maternal represents 33.4% of DALYs. Despite the progress achieved in the control of preventable childhood diseases, schistosomiasis, and the relatively low incidence of HIV/AIDS, Morocco is still facing a growing burden of infective diseases. In particular, sexually transmitted diseases and environmental related diseases such as tuberculosis, typhoid, viral hepatitis, trachoma and conjunctivitis have been persistent or increasing during the last six years [18].

Preventive measures and early detection should reduce significantly the impact of CDs and NCDs. As indicated earlier, however, the current ill-health system, with insufficient infrastructure, lack of human capacities and bad governance is unable to cope with the growing burden of disease.

## Conclusion

Morocco is facing the growing impact of communicable and non communicable diseases. In the meantime, the burden of illiteracy and the slow economic growth, conjugated to huge inequalities and sharp inequities, maintain the Human Development Index of the country at a level contrasting with its human and natural resources. The "National Initiative for Human Development" launched by the King of Morocco is based on the fact that broad fringes of the population live in precarious conditions and sometimes in a situation of poverty and marginalization contrasting with the image of Morocco. Considering the delay in human development accumulated by the country during the last decades, political initiatives are unlikely to succeed in the absence of pragmatic and efficient measures that tackle seriously the problems of regional disparities, social inequalities and health inequities.

## Dedication

This paper is dedicated to my wife Hayat and our children Wiam, Hanae and Aymane for their continuous and comprehensive family support.

## Conflict interest

The autho(r) declares that he has no competing interest.

## References

[B1] UNDP Human Development Indicators 2003. http://hdr.undp.org.

[B2] Arab Human Development Report (2002). Creating Opportunities for Future Generations. http://www.undp.org/rbas/ahdr/english2002.html.

[B3] Arab Human Development Report (2003). Building a Knowledge Society. http://www.undp.org/rbas/ahdr/english2003.html.

[B4] Arab Human Development Report (2004). Towards Freedom in the Arab World. http://www.undp.org/rbas/ahdr/english2004.html.

[B5] Gakido E, King G (2002). Measuring total health inequality: adding individual variation to group-level differences. International Journal for Equity in Health.

[B6] Heggenhougen HK (2005). The epidemiology of inequity: Will research make a difference?. Norsk Epidemiologi.

[B7] The Working Group on Health Disparities (2005). Health Disparities & the Body Politic.

[B8] Mackenbach JP, Kunst AE (1997). Measuring the magnitude of socio-economic inequalities in health: an overview of available measures illustrated with two examples from Europe. Soc Sci Med.

[B9] Sanchez P, Swaminathan MS (2005). Hunger in Africa: the link between unhealthy people and unhealthy soils. Lancet.

[B10] Boutayeb A (2005). Sustainable Development and Health Equity.

[B11] Kim JK, Mullen JV, Irwin A, Gershman J, (eds) (2002). Dying for Growth: Global Inequality and the health of the poor.

[B12] Bennis A Le système de soins de santé au Maroc. http://www.sante.gov.ma/smsm/cmm_web/Le_systeme_de_soins.htm.

[B13] Belafrej A (2003). Management and treatment of diabetic children at the University Hospital Centre in Rabat. Sante Publique.

[B14] Boutayeb A, Boutayeb S (2005). The burden of non communicable diseases in developing countries. International Journal for Equity in Health.

[B15] Boutayeb A, Twizell EH, Achouyab K, Chetouani A (2004). A mathematical model for the burden of diabetes and its complications. BioMedical Engineering Online.

[B16] Boutayeb A (2006). The double Burden of Communicable and NonCommunicable Diseases in Developing Countries. Transactions of the Royal Society for Tropical Medicine and Hygiene.

[B17] Tazi M, Abirkhalil S, Chaouki N, Cherkaoui S, Lahmouz F, Srairi J, Mahjour J (2003). Prevalence of the main cardiovascular risk factors in Morocco: results of a national survey in 2000. Journal of Hypertension.

[B18] DELM (2003). Donnees epidemiologiques des maladies sous surveillance. http://www.sante.gov.ma.

[B19] Milanovic B, Yitzhaki S (2002). Decomposing world income distribution: does the World have a middle class?. Review of Income and Wealth.

[B20] Rapport de Développement Humain PNUD Maroc (2003). Gouvernance et Accélération du Développement Humain. http://hdr.undp.org.

